# Knowledge-Based Identification of Soluble Biomarkers: Hepatic Fibrosis in NAFLD as an Example

**DOI:** 10.1371/journal.pone.0056009

**Published:** 2013-02-06

**Authors:** Sandra Page, Aybike Birerdinc, Michael Estep, Maria Stepanova, Arian Afendy, Emanuel Petricoin, Zobair Younossi, Vikas Chandhoke, Ancha Baranova

**Affiliations:** 1 Center for the Study of Chronic Metabolic Diseases, School of Systems Biology, College of Science, George Mason University, Fairfax, Virginia, United States of America; 2 Betty and Guy Beatty Liver and Obesity Program, Inova Health System, Falls Church, Virginia, United States of America; 3 Center for Liver Diseases and Department of Medicine, Inova Fairfax Hospital, Falls Church, Virginia, United States of America; 4 Center for Applied Proteomics and Molecular Medicine, School of Systems Biology, College of Science, George Mason University, Fairfax, Virginia, United States of America; 5 The Research Centre of Medical Genetics RAMS, Moscow, Russia; 6 Liver Diseases Branch of National Institute of Diabetes and Digestive and Kidney Diseases, National Institutes of Health, Bethesda, Maryland, United States of America; National Research Council of Italy, Italy

## Abstract

The discovery of biomarkers is often performed using high-throughput proteomics-based platforms and is limited to the molecules recognized by a given set of purified and validated antigens or antibodies. Knowledge-based, or systems biology, approaches that involve the analysis of integrated data, predominantly molecular pathways and networks may infer quantitative changes in the levels of biomolecules not included by the given assay from the levels of the analytes profiled. In this study we attempted to use a knowledge-based approach to predict biomarkers reflecting the changes in underlying protein phosphorylation events using Nonalcoholic Fatty Liver Disease (NAFLD) as a model. Two soluble biomarkers, CCL-2 and FasL, were inferred *in silico* as relevant to NAFLD pathogenesis. Predictive performance of these biomarkers was studied using serum samples collected from patients with histologically proven NAFLD. Serum levels of both molecules, in combination with clinical and demographic data, were predictive of hepatic fibrosis in a cohort of NAFLD patients. Our study suggests that (1) NASH-specific disruption of the kinase-driven signaling cascades in visceral adipose tissue lead to detectable changes in the levels of soluble molecules released into the bloodstream, and (2) biomarkers discovered *in silico* could contribute to predictive models for non-malignant chronic diseases.

## Introduction

One of the main goals of translational research is to identify diagnostic and prognostic biomarkers which have the potential to predict clinical outcomes. The biomarkers are proteins, protein fragments, or other molecules produced by the body in response to the presence of ailing tissue or by the diseased tissue itself. Ideally, these biomarkers should be accessible in a minimally invasive way through assaying the serum, urine or other body fluids and tissues. More often than not, initial exploratory studies aimed at the discovery of biomarkers are performed using high-throughput proteomics-based platforms, for example, the label-free mass spectrometry [1], the antibody arrays [2] and the reverse phase protein arrays [3]. The high-throughput approach described above is also known as so-called “unbiased” approach as it may uncover truly novel biomarker molecules reflecting yet unknown pathological mechanisms for given condition [4]. This, most strong, advantage of “unbiased” strategies is mirrored by its weakness as lack of bias comes in hand with its lack of connection to known pathophysiological processes, thus, being bound to produce at least some spurious results.

One way around this issue is to use knowledge-based, or systems biology approaches that involve the analysis of integrated data, predominantly, molecular pathways and networks already known to be implicated in given condition [4], [5]. If the systems biology model in use truly reflects the real state of the organism of study, one should be able to infer quantitative changes in the levels of biomolecules not included by the given assay from the levels of the analytes profiled. However desirable, this level of state-of-the-art detection has not been achieved yet most likely due to an inherent complexity of biological samples and the high level of technical and biological noise present in clinical samples.

In this study we attempted to use a knowledge-based approach to predict biomarkers reflecting the changes in the protein phosphorylation events profiled in a high-throughput manner. As a model, we chose Nonalcoholic Fatty Liver Disease (NAFLD) which is the most common form of chronic liver disease in the U.S. and worldwide [6]. In some patients, NAFLD manifests as simple steatosis, however, other may develop nonalcoholic steatohepatitis (NASH) that, in turn, may progress to NASH-related cirrhosis, and hepatocellular carcinoma [6].

At present, liver biopsy remains an imperfect “gold standard” of assessing whether patient has NAFLD, to diagnose NASH or to stage the extent of fibrosis. As histologic lesions of NASH may not be evenly distributed throughout the liver parenchyma, the sampling errors are to be expected [7]. Despite recent technological improvements (e.g., automatic biopsy guns, ultrasound guidance), liver biopsy remains costly, and is associated with some potentially important complications [7]. To overcome the drawbacks of liver biopsy, alternative non-invasive methods for diagnosing NAFLD have been developed [8]. These methods range from serum biomarker assays to advanced imaging techniques [9], [10]. Currently, the use of non-invasive diagnostic procedures for liver conditions is recommended as pre-screening tool, which may allow physicians to stratify the patients’ population before definitive testing by biopsy of the liver [10]. In addition, non-invasive biomarkers adequately reflecting the health and the pathological processes within the liver may be utilized to monitor the safety of the non-NAFLD related drugs metabolized by the liver. Another important consideration for the development of NAFLD biomarkers is the lack of efficient therapy for this condition. Such biomarkers would facilitate selection of drug candidates and the studies of their mechanisms of action, and enable clinical trials to be shortened and run with reduced sample size. Efficient development of non-invasive biomarkers of NAFLD, NASH and liver fibrosis requires tight collaboration between experimental and computational scientists, database experts, physicians and regulatory institutions.

One requirement for the biomarker that fits this description is that it should be soluble, be present in the bloodstream at substantial concentrations and be quantifiable by antibody-based assays. A set of soluble adipokines released by adipocytes as well as other adipose-resident cells, including macrophages, is known to play an important role in pathogenesis of the diseases of NAFLD spectrum [11], [12], thus satisfying both the NAFLD biomarker requirements outlined above and the knowledge-based discovery principle.

In this study, we sought to apply knowledge-based algorithms to determine (1) whether NASH-specific disruption of the kinase-driven signaling cascades in visceral adipose tissue leads to detectable changes in the levels of adipokines and cytokines released into the bloodstream, and (2) whether biomarkers discovered *in silico* could contribute to predictive models for NASH and NASH-related fibrosis. Overall, our study outlines an efficient strategy for the identification of non-invasive biomarkers for NAFLD and other chronic diseases.

## Materials and Methods

### Knowledge-based Enrichment Analysis

Enrichment analysis of the phosphoproteomic dataset previously described in Younossi *et al* [*13*] was conducted using MetaCore software (GeneGo, Inc.). The analysis presented in Younossi *et al.* aimed to identify the insulin-mediated pathways most represented by the collection of phosphoproteins tested and to highlight which signaling molecules exhibited the greatest differences in phosphorylation between patients with and without NASH, based on statistical modeling. The input data for enrichment analysis consisted of relative fluorescent unit values derived from reverse phase protein array dataset [13]. These values were compared between patients with and without NASH in pairwise fashion using the “common pathway” option and a significance cutoff of p<0.05.

### Pathway Tools

**Figure 1:**
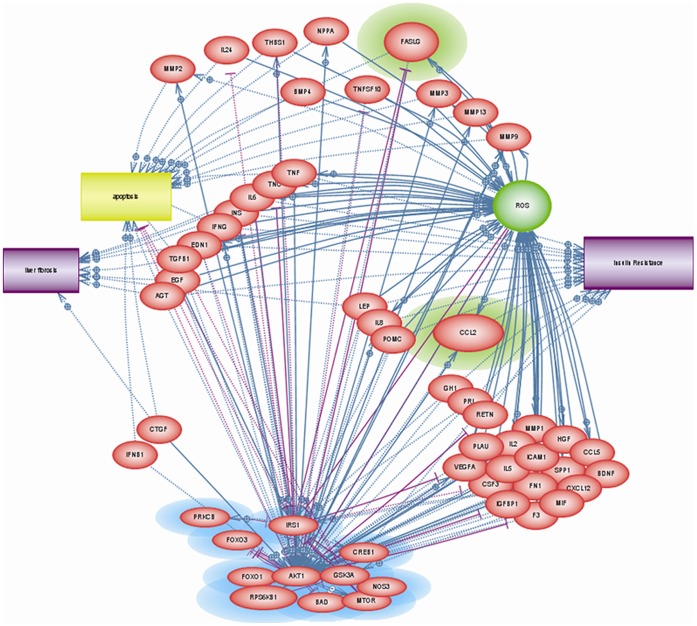
Final output network based on analyses performed in Pathway Studio. Proteins highlighted in blue were differentially phosphorylated in the phosphoproteomic data set.^24^ Proteins selected for testing in the NASH and NASH-related fibrosis biomarker panel are highlighted in green (Fas ligand and CCL2).

Pathway Studio (Ariadne Genomics) was used to systematically search the current body of literature for proteins secreted in response to altered insulin signaling. To reveal associations between previously described significant predictors of NASH [13] and the processes central to NAFLD pathogenesis, the “enrichment analysis,” “shortest path,” and “add neighbors” options were utilized. Statistical significance for results of pairwise analysis was assessed by Mann Whitney U test. A series of networks containing differentially expressed molecules was generated, and the networks containing the most differentially expressed phosphoproteins were selected for subsequent analysis. Diseases and cellular processes pertinent to NASH, specifically liver fibrosis, insulin resistance, apoptosis, and reactive oxygen species (ROS) were added as outcome categories. The resulting molecular network was then culled to a manageable level as follows: (1) we retained only those molecules with a direct relationship (*i.e.* causal or regulatory, as supported by laboratory evidence) or a nearly direct relationship (*i.e.* having a second degree connection between a molecule and an object (disease process, etc.) or a first degree relationship that is derived from correlative laboratory data from several independent sources) to the phosphoproteins from the study of Younossi *et al.* or to the outcome categories; (2) we deleted relationships based on scant evidence (*e.g.* only one publication supporting a relationship); and (3) we retained only soluble molecules, favoring small peptides over other types of secreted molecules such as steroid hormones. The final network resulting from this “culling” step is depicted at Figure 1.

### Patient Cohort for Validation Study

The study was conducted in ongoing collaboration with the Center for Liver Diseases at Fairfax INOVA Hospital (Falls Church, VA). After informed consent, human serum samples were collected by the Center from patients with histologically proven NAFLD, stripped of traceable identifiers and placed into a −80°C repository. Each sample was accompanied by a core liver biopsy description read by a single hepatopathologist, who used a standardized approach to stage and grade each sample for steatosis, NASH, and fibrosis that was assessed with the Masson trichrome stain. Portal fibrosis and interlobular pericellular fibrosis were assessed separately and graded as follows: 0 =  none; 1 =  mild; 2 =  moderate; and 3 =  marked. Biopsies of the fibrosis-negative group of patients were scored 0 for both kinds of fibrosis, portal or pericellular; biopsies of the fibrosis-positive group of patients had mild to moderate pericellular or portal fibrosis, or both; patients with advanced fibrosis with at least moderate portal or pericellular fibrosis, bridging fibrosis, or cirrhosis. NASH was defined as steatosis, lobular inflammation, and ballooning degeneration with or without Mallory-Denk bodies and/or fibrosis. Clinical and demographic data were available for all 37 patients. The study protocol was approved by the Institutional Review Board of Fairfax INOVA Hospital.

### ELISAs

Serum levels of CCL-2 were measured by the Human CCL2/MCP-1 Immunoassay Quantikine ELISA kit, while serum concentrations of sFasL were assessed using the Human Fas Ligand/TNFSF6 Quantikine ELISA kit; both from R & D Systems (Minneapolis, MN, USA). All measurements were performed in duplicate and the manufacturer’s instructions were followed. Absorbance readings at 450 nm were made using an ELx800 plate reader. Wavelength correction was performed by subtraction of readings at 630 nm. Calibration (standard) curves were constructed and curve fitting was conducted as specified by the manufacturer’s protocol. Concentrations of analyte in each sample were calculated from the standard curve using Gen5 software and the average of each duplicate pair of measurements was used in subsequent analyses.

### Statistical Analysis

The entire study cohort was divided into sub-cohorts according to the following diagnostic comparisons: (1) those with NASH were compared to those without NASH; (2) those with any hepatic fibrosis were compared to those with no fibrosis; and (3) those with advanced hepatic fibrosis were compared to those with minimal to no fibrosis. Each of these cohorts was further analyzed separately by calculating the means and standard deviations for all continuous variables and counts and percentages for all categorical variables. Then, two-sample statistical tests were performed for each parameter in each comparison. To choose an appropriate statistical test, all continuous variables were tested for normality by the Shapiro Wilk test; a p-value ≤0.05 was considered significant and thus indicative that the data came from a non-normally distributed population. For normally distributed data comparisons between groups were made by two-tailed, two-sample t-test assuming separate (unpooled) variances and for non-normally distributed data comparisons between groups were made by Mann-Whitney U (Wilcoxon rank sum) test. For categorical variables, group comparisons were made using the Pearson chi-square test for homogeneity except in cases where at least one cell count was <5; in those cases a Fisher’s exact test was performed. Multiple linear and logistic regressions with stepwise, bidirectional selection was used to develop predictive models for the occurrence of NASH, hepatic fibrosis and advanced hepatic fibrosis. Since the study cohort was small, to avoid over-fitting the model, only the following fifteen variables were tested as independent predictors: gender, race, age, BMI, diabetes, hyperlipidemia, AST, ALT, total bilirubin, glucose, total cholesterol, triglycerides, HDL, CCL-2, and FasL. A model having an overall p-value of ≤0.05 was considered significant, even if some predictors within the model were above that threshold. Tests of normality, parametric- and non-parametric tests were performed using Mystat: a Student Version of Systat (v.12). Multiple linear and logistic regressions, chi square- and Fisher’s Exact tests were performed using S-Plus (v.8), and the ROC (receiver operating characteristic) analyses were generated with the MedCalc statistical tool.

The predictive performance was evaluated for the generated models using the sensitivity, specificity, and area under the ROC-curve (AUC) with 95 percent confidence intervals (CIs) characteristics. The logistic regression models were cross-validated with the leave-one-out (LOO) method that determines how accurate the learning algorithm is by training it multiple times using all but one of the training set data points.

## Results

### Knowledge-Based Enrichment Analysis

**Figure 2:**
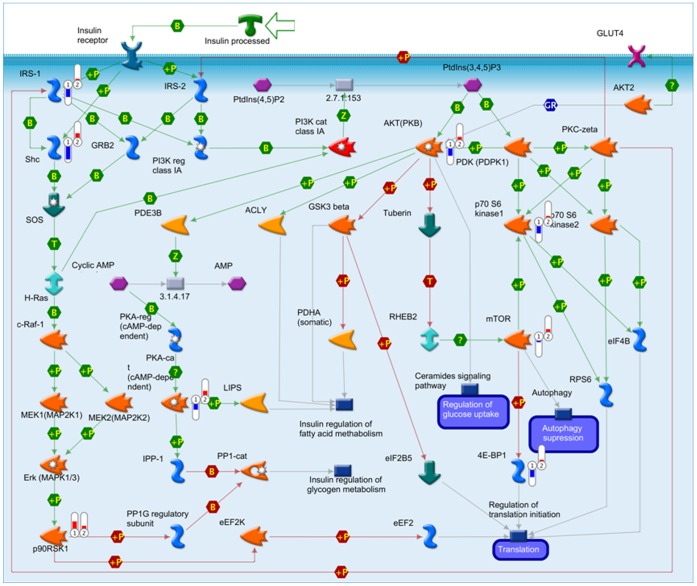
MetaCore output showing regulation of lipid metabolism by insulin. Relative phosphorylation levels of proteins measured in Younossi *et al* are indicated by bars (bar 1 =  patients with NASH; bar 2 =  patients without NASH). Bars point up (red) or down (blue) in relation to the assay normalization value; bar height indicates the degree of difference in phosphorylation from the normalization value.

Enrichment analysis revealed that the top insulin-mediated pathways most enriched in the Younossi *et al.* [13] dataset were the IGF-1 receptor pathway, insulin regulation of translation, AKT signaling, PIP3 signaling and regulation of lipid metabolism by insulin. Table 1 shows which of the original 27 proteins analyzed appeared in those five pathways. The enrichment analysis also highlighted which components of these pathways were most differentially phosphorylated in patients with and without NASH. For example, in the regulation of lipid metabolism by insulin, the proteins most differentially phosphorylated were insulin receptor substrate 1 (IRS-1) and its binding partner, SHC-transforming protein (SHC), and to a lesser extent 3-phosphoinositide dependent protein kinase-1 (PDPK1), ribosomal protein S6 kinase, 70 kDa (p70-S6 kinase 2), and eIF4E-binding protein 1 (4E BP1). In all of these cases phosphorylation levels were notably lower in patients with NASH relative to those without NASH, whereas other measured proteins in this pathway (*e.g.* mechanistic target of rapamycin (mTOR)) were not as strikingly different in their phosphorylation levels (Figure 2). In fact, several proteins were consistently non-phosphorylated in the insulin-mediated pathways evaluated and therefore it is not surprising that a subset of them (IRS-1, AKT) were independently predictive of NASH and NASH-related fibrosis in regression models [13].

**Table 1:** Subset of phosphoproteins analyzed in Younossi *et al.* ^8^ appearing in the five most enriched pathways.

Phosphoprotein	Function	Pathway(s)
IRS-1	docking protein	regulation of lipid metabolism by insulin
		signal transduction by AKT
		signal transduction by PIP3
		IGF-1 receptor signaling
		regulation of translation by insulin
SHC	protein domain	regulation of lipid metabolism by insulin
		signal transduction by PIP3
		IGF-1 receptor signaling
		regulation of translation by insulin
AKT	kinase	regulation of lipid metabolism by insulin
		signal transduction by AKT
		signal transduction by PIP3
		IGF-1 receptor signaling
		regulation of translation by insulin
P70-S6	kinase	regulation of lipid metabolism by insulin
		signal transduction by AKT
		signal transduction by PIP3
		IGF-1 receptor signaling
		regulation of translation by insulin
PKA-c	kinase	regulation of lipid metabolism by insulin
mTOR	kinase	regulation of lipid metabolism by insulin
		signal transduction by PIP3
		IGF-1 receptor signaling
		regulation of translation by insulin
4E-BP1	translation	regulation of lipid metabolism by insulin
		signal transduction by AKT
		signal transduction by PIP3
		IGF-1 receptor signaling
		regulation of translation by insulin
p90 RSK1	kinase	regulation of lipid metabolism by insulin
		signal transduction by PIP3
		IGF-1 receptor signaling
		regulation of translation by insulin
GSK-3	kinase	signal transduction by AKT
		signal transduction by PIP3
		IGF-1 receptor signaling
FOXO3A	transcription factor	signal transduction by AKT
		signal transduction by PIP3
		IGF-1 receptor signaling
BAD	apoptosis	signal transduction by AKT
		signal transduction by PIP3
		IGF-1 receptor signaling
CREB1	transcription factor	signal transduction by PIP3
		IGF-1 receptor signaling
eIF4G	translation	regulation of translation by insulin

### Identification of Soluble Proteins Associated with Insulin Signaling

Pathway Studio guided literature searches were performed to establish (1) whether the biological mechanisms of action for the soluble molecules highlighted by this analysis are relevant to NASH and NASH-related fibrosis; and (2) whether any previous associations had been made between a protein of interest and the occurrence of NASH or NASH-related fibrosis. Of the more than 50 proteins that fit the loose criteria for molecules of interest, several cytokines (*e.g.* TNFα, IL-6, IL-8) were excluded from further consideration, having been previously measured by our and other laboratories and found to lack differentiating power in context of NASH and NASH-related fibrosis [10], [12]. Others were excluded from consideration based on their direct association with specific processes (insulin, IFNα, etc.). Fas ligand (FasL) and Chemokine (C-C motif) ligand 2 (CCL-2) were ultimately selected for testing because at the time our study was undertaken, neither had been tested as biomarkers of NAFLD or NASH, yet well-described in term of their biological functions and known for their pleiotropy. The detailed rationale for the selection of CCL-2 and FasL molecules for further validation studies is in Information S1.

### Validation of the Soluble Biomarkers Predicted in silico

To find out whether soluble biomarkers inferred from the NASH-associated changes in the adipose tissue’ phosphoproteome using a knowledge-based *in silico* approach, the levels of FasL and CCL-2 were measured in 37 serum samples previously collected in the course of the ongoing study of NAFLD pathogenesis.

### Performance of FasL and CCL-2 in the Prediction of NASH

Demographic, clinical, laboratory and histological data and the outcomes of group statistical comparisons for patients with and without NASH are listed in Table 2. No clinical or demographic attributes were statistically different in patients with NASH as compared to those without NASH, except for hepatic fibrosis, which was significantly more prevalent in NASH patients (p<0.015). Serum levels of both candidate biomarkers, CCL-2 and sFasL, were not different between NASH and non-NASH cohorts. Respectively, multiple linear regression using bi-directional stepwise selection revealed that for this patient cohort, only BMI and HDL were independent predictors for NASH (p<0.048), while the logistic regression analysis cross-validated using Leave-One-Out algorithm confirmed this model with p<0.0218 (Table 3).

**Table 2:** Demographic, clinical, and laboratory data for patients with and without NASH.

	*NASH*	*no NASH*	*P-value*	*Test*
N	22	15		
Fibrosis (any)	22 (100%)	10 (67%)	**0.015**	Chi
Advanced fibrosis	3 (14%)	3 (20%)	0.667	FE
Diabetes	8 (36%)	5 (33%)	0.872	Chi
Female	16 (73%)	9 (60%)	0.647	Chi
Caucasian	19 (86%)	12 (80%)	0.951	Chi
Age	49±9	47±11	0.596	2T
BMI	49±11	46±9	0.421	MW
Hyperlipidemia	12 (54%)	11 (73%)	0.417	Chi
Hypertension	15 (68%)	11 (73%)	0.801	Chi
AST (U/L)	23±6.4	22±7.4	0.760	2T
ALT (U/L)	35±18	29±9	0.496	MW
AST: ALT	0.74±0.21	0.82±0.34	0.577	MW
Albumin (g/dL)	4.1±0.27	3.9±0.77	0.732	MW
Bilirubin (total) (mg/dL)	0.44±0.17	0.59±0.38	0.263	MW
White blood cell count(10^3^/uL)	7.6±2.2	6.9±1.6	0.246	MW
Platelet count (10^3^/uL)	274±78	270±69	0.845	2T
Hemoglobin (g/dL)	13±1.1	13±1.7	0.878	2T
Glucose (mg/dL)	116±43	104±32	0.556	MW
Cholesterol (total) (mg/dL)	187±30	190±41	0.808	2T
Triglycerides (mg/dL)	179±144	174±83	0.458	MW
HDL (mg/dL)	47±9	51±11	0.182	2T
CCL-2 (pg/mL)	464±118	486±218	0.902	MW
sFasL (pg/mL)	89±31	82±34	0.516	MW
Portal fibrosis	16 (73%)	10 (67%)	0.976	Chi
Pericellular fibrosis	12 (54%)	0 (0%)		*n/a*

Entries are counts for discrete measures (with percentage of group total given in parentheses) or mean ± S.D. for continuous measures. A p-value of ≤0.05 was considered significant. Significant results are shown in bold text. Chi = chi square test of homogeneity; FE = Fisher’s exact test; MW = Mann-Whitney U test; 2T = two-sample t-test (2-tailed).

**Table 3:** Model for the prediction of NASH.

	*Odds ratio*	*CI 95%*	*p-value*
(Intercept)	0.856	0.009–77.801	0.9460
BMI	0.847	0.724–0.991	0.0377
HDL (mg/dL)	1.167	1.028–1.324	0.0222

Cross-validation of this model adjusted p value to less than 0.0218 with following characteristics: AUC: 0.709 (CI: 0.537–0.846), Optimal sensitivity: 63.64 (CI: 40.7–82.8); Optimal specificity: 86.67 (CI: 59.5–98.3); Cut-off: OR = 1.56.

### Performance of FasL and CCL-2 in the Prediction of Fibrosis

None of the demographic and clinical variables were significantly different between patients with and without fibrosis (Table 4), however, 69% of patients with fibrosis also had NASH and 19% of patients with fibrosis had advanced fibrosis. sFasL levels were significantly higher (p<0.015) in patients with fibrosis relative to those without fibrosis, while CCL-2 levels were not different. By multiple linear regression using stepwise bidirectional selection, both candidate biomarkers, CCL-2 and sFasL, as well as race were independent predictors of hepatic fibrosis (p<0.007). The logistic regression analysis cross-validated using Leave-One-Out algorithm confirmed this model with p<0.0134 (Table 5).

**Table 4:** Demographic, clinical, and laboratory data for patients with and without any hepatic fibrosis.

	*Fibrosis*	*no Fibrosis*	*P-value*	*Test*
N	32	5		
NASH	22 (69%)	0 (0%)		*n/a*
Advanced fibrosis	6 (19%)	0 (0%)		*n/a*
Diabetes	10 (31%)	3 (60%)	0.321	FE
Female	23 (72%)	2 (40%)	0.304	FE
Caucasian	28 (88%)	3 (60%)	0.177	FE
Age	48±10	49±11	0.847	2T
BMI	48±10	47±13	0.564	MW
Hyperlipidemia	19 (59%)	4 (80%)	0.630	FE
Hypertension	23 (72%)	3 (60%)	0.603	FE
AST (U/L)	22±6.4	22±10	0.956	2T
ALT (U/L)	33±16	30±6.9	0.807	MW
AST: ALT	0.77±0.21	0.81±0.56	0.548	MW
Albumin (g/dL)	4.0±0.56	4.1±0.34	0.806	MW
Bilirubin (total) (mg/dL)	0.51±0.29	0.44±0.23	0.667	MW
White blood cell count(10^3^/uL)	7.3±1.9	7.4±2.3	0.773	MW
Platelet count (10^3^/uL)	267±72	306±79	0.347	2T
Hemoglobin (g/dL)	14±1.3	13±1.3	0.299	2T
Glucose (mg/dL)	112±41	103±17	0.947	MW
Cholesterol (total) (mg/dL)	191±34	170±35	0.267	2T
Triglycerides (mg/dL)	182±126	142±87	0.351	MW
HDL (mg/dL)	48±8.9	51±16	0.667	2T
CCL-2 (pg/mL)	457±138	570±279	0.374	MW
sFasL (pg/mL)	91±30	54±26	**0.015**	MW
Portal fibrosis	26 (81%)	0 (0%)		*n/a*
Pericellular fibrosis	12 (38%)	0 (0%)		*n/a*

Entries are counts for discrete measures (with percentage of group total given in parentheses) or mean ± S.D. for continuous measures. A p-value of ≤0.05 was considered significant. Significant results are shown in bold text. FE = Fisher’s exact test; MW = Mann-Whitney U test; 2T = two-sample t-test (2-tailed).

**Table 5:** Model for the prediction of any hepatic fibrosis.

	*Odds ratio*	*CI 95%*	*p-value*
(Intercept)	72.617	0.165-31914.126	0.1675
Caucasian	0.0006	<0.001–6.194	0.1483
CCL-2 (pg/mL)	1.022	0.995–1.049	0.1159
sFasL (pg/mL)	0.821	0.665–1.012	0.0647

Cross-validation of this model adjusted p value to less than 0.0134 with following characteristics: AUC: 0.750 (CI: 0.581–0.877), Optimal sensitivity: 59.38 (CI: 40.6–76.3); Optimal specificity: 80.00 (CI: 28.4–99.5); Cut-off: OR = 6.29.

### Performance of FasL and CCL-2 in the Prediction of Advanced Fibrosis

In our study, two diagnostic groups (advanced fibrosis *vs.* minimal to none) could not be distinguished by demographic factors. However, several clinical variables were different when these two groups were compared, namely, AST, ALT and HDL serum levels (Table 6). Interestingly, despite the fact that serum levels of the candidate biomarkers CCL-2 and sFasL were not different between cohorts, an analysis by multiple linear regression showed that CCL-2, along with HDL levels, were independent predictors of advanced hepatic fibrosis (p<0.028). The logistic regression analysis cross-validated using Leave-One-Out algorithm confirmed this model with p<0.0037 (Table 7).

**Table 6:** Demographic, clinical, and laboratory data for patients with and without advanced fibrosis.

	*Adv. Fibrosis*	*none to minimal Fibrosis*	*P-value*	*Test*
N	6	31		
NASH	3 (50%)	19 (61%)	0.667	FE
Diabetes	3 (50%)	10 (32%)	0.664	FE
Female	4 (67%)	21 (68%)	1.00	FE
Caucasian	5 (83%)	26 (84%)	0.561	Chi
Age	51±10	48±10	0.483	2T
BMI	48±2.6	48±11	0.564	MW
Hyperlipidemia	4 (67%)	19 (61%)	1.00	FE
Hypertension	6 (100%)	20 (65%)	0.244	Chi
AST (U/L)	20±2.4	23±7.2	**0.042**	2T
ALT (U/L)	23±4.7	34±16	**0.026**	MW
AST: ALT	0.88±0.15	0.75±0.29	0.122	MW
Albumin (g/dL)	4.0±0.28	4.0±0.57	0.804	MW
Bilirubin (total) (mg/dL)	0.52±0.28	0.49±0.28	0.753	MW
White blood cell count (10^3^/uL)	8.0±2.7	7.2±1.8	0.592	MW
Platelet count (10^3^/uL)	256±99	276±69	0.649	2T
Hemoglobin (g/dL)	12.4±1.8	13.6±1.2	0.156	2T
Glucose (mg/dL)	103±41	113±39	0.606	MW
Cholesterol (total) (mg/dL)	203±15	185±36	0.065	2T
Triglycerides (mg/dL)	144±39	183±131	0.853	MW
HDL (mg/dL)	56±7.8	47±9.8	**0.041**	2T
CCL-2 (pg/mL)	390±103	488±169	0.161	MW
sFasL (pg/mL)	86±33	86±33	0.805	MW
Portal fibrosis	5 (83%)	21 (68%)	0.782	Chi
Pericellular fibrosis	3 (50%)	9 (29%)	0.367	FE

Entries are counts for discrete measures (with percentage of group total given in parentheses) or mean ± S.D. for continuous measures. A p-value of ≤0.05 was considered significant. Significant results are shown in bold text. Chi = chi square test of homogeneity; FE = Fisher’s exact test; MW = Mann-Whitney U test; 2T = two-sample t-test (2-tailed).

**Table 7:** Model for the prediction of advanced fibrosis.

	*Odds ratio*	*CI 95%*	*p-value*
**(Intercept)**	130.817	0.331–51647.238	0.1101
**HDL**	0.879	0.782–0.987	0.0299
**CCL-2**	1.008	0.999–1.017	0.0933

Cross-validation of this model adjusted p value to less than 0.0037 with following characteristics: AUC: 0.750 (CI: 0.581–0.877), Optimal sensitivity: 59.38 (CI: 40.6–76.3); Optimal specificity: 80.00 (CI: 28.4–99.5); Cut-off: OR = 0.18.

## Discussion

Inferring clinically relevant insights from the complex picture of the quantitative changes in expression levels and post-translational modifications of proteins remains a major challenge in systems biology. The most crucial point in this process is the interpretation of the disease signature that includes molecular changes that might be causal, associated with or incidental to the observed phenotype. Application of knowledge-based algorithms can produce reasonable hypotheses linking altered pathways to phenotypic changes. However, even the causally proven connections between an observed change and the disease phenotype itself often lacks practical applicability for diagnostics, either due to the expensive nature of the technology used for initial high-throughput profiling or to the necessity of invasive probing for the collection of the diseased tissue. The idea to take knowledge-based algorithms one step further and use them for the inference of soluble and, therefore, easily accessible, molecules that change levels as a result of the disturbance of certain regulatory pathways central to a given pathological condition, is tempting, but, until now, remains untested.

This study utilizes the statistical tools and knowledge base of two pathway analysis packages applied to previously generated data from our laboratory to predict molecules likely to be differentially expressed by disease, and then measures the expression of these molecules. Our goal was to demonstrate that knowledge-based algorithms may augment biomarker research. As the test case, we chose Nonalcoholic Fatty Liver Disease (NAFLD), the disease with the prevalence estimated as high as 30% in the U.S. adult population, 20% in the non-U.S. adult population, and 2.6% in the U.S. pediatric population [6].

In previous studies, the involvement of adipose-derived soluble molecules, adipokines and cytokines, in the pathology of NAFLD was clearly demonstrated [11], [12]. Previously performed multiplexed phosphoprotein signaling study in adipose tissue samples profiled 54 different kinase substrates and cell signaling endpoints and showed a suppression of insulin signaling in visceral adipose tissue from patients with NASH [13]. The phosphorylation states of some of these proteins, namely AKT1 and IRS1, in combination with clinical and demographic parameters were shown to be independent predictors of NASH [13]. The most crucial limitation of this study was that, despite the predictive power uncovered, the use of the adipose–based protein phosphorylation patterns would require a fairly invasive tissue biopsy, while a serum based diagnostic tool would be a far more facile way of monitoring NAFLD disease pathogenesis. Therefore, we attempted to use *in silico* network analysis to predict soluble biomarker candidates reflecting the changes in the phosphoprotein signaling previously observed in tissue samples of NASH patients.

To this end, we performed enrichment analyses and selected a number of secreted molecules could be linked to these pathways and to NAFLD-associated disease processes. A shortlist of these molecules included candidate serum biomarker CCL-2/MCP-1 and sFasL. As guided by knowledge-based algorithms, we hypothesized that (1) circulating CCL-2 levels should be elevated in patients with NAFLD due to an increase in its secretion from enlarged visceral adipose tissue and as evident by monocyte infiltration into the NAFLD livers; and (2) deregulated insulin signaling in adipose tissue increases Fas/FasL expression and its release into the bloodstream that promote an apoptosis of hepatocytes. The cell death and the inflammation within the liver would then be expected to promote fibrogenesis as part of the liver’s healing response.

Importantly, the CCL-2/MCP-1 and sFasL potentially belong to two different classes of biochemical biomarkers. sFasL is predicted to behave as core biomarker, in that it should directly contribute to the deregulated hepatocytic cell death, while CCL-2/MCP-1 reflects systemic inflammation background that underlies the diseases of NAFLD spectrum [14]. Interestingly, the results of this study suggest that both candidate biomarkers are potentially useful for the prediction of hepatic fibrosis. Surprisingly, serum levels of CCL-2 decreased rather than increased with disease severity and CCL-2 was a significant predictor of fibrosis even after controlling for sFasL and race, and a significant predictor of advanced fibrosis after controlling for HDL. This trend was unexpected given that another study reported higher serum levels of CCL-2 in patients with NAFLD relative to controls and in patients with NASH compared to those with simple steatosis [14]. In our study, the average serum concentrations of CCL-2 decreased with disease severity, such that in patients diagnosed with steatosis, NASH, fibrosis (with or without NASH), and advanced fibrosis, average serum concentrations were 570, 464, 457, and 445 pg/mL, respectively. One possible explanation to an inverse relationship between the levels of CCL-2 and the severity of chronic liver disease is that the metabolic activity of CCL-2 responsive neutrophils and other infiltrating white blood cells diminishes together with the intensification of liver failure [15].

In contrast, serum levels of sFasL were significantly higher in patients with any form of hepatic fibrosis compared to those without it and increasing sFasL was a significant predictor of hepatic fibrosis even after controlling for race and CCL-2. Serum levels of sFasL were not significantly different between patients with and without NASH or between patients with and without advanced fibrosis. Similarly, sFasL was not a component of any significant model predicting NASH and advanced fibrosis. In studies of non-NAFLD liver diseases, sFasL has been used as a biomarker of hepatocytic apoptosis with some success. Elevated serum levels of sFasL distinguished patients with acute liver failure from those with acute hepatitis E or with sepsis alone [16]. Serum levels of sFasL were also higher in patients with chronic but not acute hepatitis B and were highest in patients with the greatest degree of infection [17]. Likewise, serum levels of sFasL were elevated in patients with alcohol-related cirrhosis and it was subsequently shown that peripheral blood mononuclear cells (PBMCs) of patients with alcohol-related cirrhosis secreted higher levels of sFasL relative to controls [18].

Our study indicates that the levels of sFasL and CCL-2 are reflective of the degree of liver fibrosis rather than the inflammatory features of NASH. The monitoring of the fibrotic changes in the liver is important part of the monitoring and therapy for many non-NAFLD conditions, for example, for chronic hepatitis C, for alcoholism, for the patients undergoing rapid weight loss that is shown to cause transient spikes in levels of liver enzymes, portal inflammation and fibrosis [19], [20]. In our opinion, the fibrosis biomarker properties of CCL-2 and sFasL warrants further investigation and validation as they may augment current clinical practice through their combination with existing non-invasive liver fibrosis tests, for example, APRI [10].

A drawback to the current study is the small size of our validation cohort of 37 subjects. Lack of statistical power may explain why the target biomarkers were not useful for predicting NASH and why sFasL was an independent predictor of fibrosis but not advanced fibrosis. Future testing of CCL-2 and sFasL should therefore be conducted on a larger study cohort with more examples of discrete diagnostic categories; *e.g.* more examples of patients with steatosis but no inflammation might help to elucidate the use of CCL-2 as a biomarker, since CCL-2 has been speculated to play a role in the transition from simple steatosis to NASH [14]. Future experiments should include simultaneous measurements of other endpoints that might lend credence to the hypothesized actions of our biomarkers. For example, it would be wise to measure established biomarkers of apoptosis (*e.g.* caspase-cleaved cytokeratin 18) concurrently with sFasL so that correlations between the two can be calculated; a positive correlation would underscore the role of sFasL as a biomarker of apoptosis in the context of NAFLD. Additionally, to enhance the performance of known non-invasive biomarkers of liver fibrosis, there may be a value in combining them with CCL-2 and sFasL in a comprehensive panel. Another important avenue for future investigation is to find out whether CCL-2 and sFasL specifically reflect a severity of the liver disease, or rather correlate with some other pathophysiological aspects of being obese and insulin resistant, for example, of the systemic inflammation or the co-morbid conditions.

While an introduction of CCL-2 and FasL into clinical practice of NAFLD diagnostics may not be immediate, our study demonstrates that the pathway analysis technologies may hold their promise to make the process of *in silico* biomarker discovery more systematic and customizable. In our study, the utility of knowledge-based analysis for the inference of serum biomarker candidates was demonstrated for non-malignant conditions, thus, indicative of the potential of this approach for translational research.

### Conclusions

Here we described an attempt to use a knowledge-based approach to predict biomarkers reflecting the changes in the protein phosphorylation events profiled in a high-throughput manner using Nonalcoholic Fatty Liver Disease (NAFLD) as a model. Two soluble biomarkers, CCL-2 and sFasL, were identified *in silico* as relevant to NAFLD pathogenesis. Predictive performance of these biomarkers was studied using serum samples collected from patients with histologically proven NAFLD. Our approach to biomarker discovery, in which we used pathway-oriented informatics and applied knowledge databases to make predictions about the progression of NAFLD, is important as it could be easily replicated for any disease process involving multiple organs or tissues.

## Supporting Information

Information S1 **Detailed Pathway Studio Guided Rationale for the Selection of CCL-2 and FasL Molecules for Further Validation Studies.**
(DOCX)
