# Supplementary material for: Knowledge-Based Identification of Soluble Biomarkers: Hepatic Fibrosis in NAFLD as an Example
Source: PLoS One. 2013 Feb 6;8(2):e56009. doi: 10.1371/journal.pone.0056009 (PMC3566090; doi:10.1371/journal.pone.0056009)
Supplement: Information S1 — Detailed Pathway Studio Guided Rationale for the Selection of CCL-2 and FasL Molecules for Further Validation Studies. (DOCX) [file pone.0056009.s001.docx]

**Information S1.**

**Detailed Pathway Studio Guided Rationale for the Selection of CCL-2 and FasL Molecules for Further Validation Studies.**

***CCL-2.***

Also known as monocyte chemotactic protein-1 (MCP-1), CCL-2 belongs to the CC chemokine family and is encoded by a gene located on the q-arm of chromosome 17. At sites of inflammatory response, CCL-2 is a chemoattractant for monocytes and memory-phenotypic T-lymphocytes and induces the migration of dendritic cells [1, 2]. CCL-2 is a ligand for the G-protein coupled receptor CCR2 and triggers signaling cascades that lead to increased cytosolic Ca2+ ion concentrations, stimulation of the MAPK series of kinases and activation of Rho GTPases in monocytes [2-4]. It contributes to various healing processes ranging from the reduction of viral infection to bone remodeling [5]. Elevated expression of CCL-2 is associated with a number of diseases with inflammatory components including multiple sclerosis, rheumatoid arthritis, atherosclerosis, inflammatory bowel disease and cancer [5, 6].

CCL-2 is secreted by many cell types (e.g. peripheral blood mononuclear cells (PBMC’s), mast cells, epithelial cells, endothelial cells, and smooth muscle cells) and its secretion is induced by inflammatory mediators (e.g. interleukin-1 alpha (IL-1α), interferon-gamma (IFN-γ)) [7-11].

Inflammation of the liver is a hallmark of NAFLD, however systemic inflammation may also be important in the etiology or progression of the disease given the strong association between NAFLD and obesity [12] and between obesity and circulating inflammatory factors [13]. The systemic inflammation associated with obesity has been attributed to the secretion of pro-inflammatory cytokines from both adipocytes and macrophages embedded in adipose tissue. CCL-2 is one of these cytokines and its secretion is higher in obese subjects [14]. Moreover, rodent model studies suggest that CCL-2 may play a mechanistic role in the etiology of NAFLD [15, 16].

Taken together, these data prompted us to hypothesize that, in patients with NAFLD, circulating CCL-2 should be elevated due to its increased secretion from enlarged visceral adipose tissue compartments. Increased levels of CCL-2 may cause monocyte infiltration into the liver and, thus, contribute to steatosis and hepatic insulin resistance, therefore being reflective of one or more of the histological features of NAFLD.

***Fas Ligand.***

Also known as TNF superfamily member 6 (TNFSF6), CD178 antigen and CD95 ligand, Fas ligand (FasL), is a member of the tumor necrosis factor (TNF) superfamily. FasL is a single-pass, type II transmembrane protein consisting of 281 amino acids. Binding of FasL to its receptor, Fas, a type I transmembrane protein, induces apoptosis. The canonical extrinsic pathway for FasL signaling includes oligomerization of the Fas upon its binding, recruitment of the fas-associated death domain (FADD) protein followed by binding to pro-caspase-8 and formation of the death-inducing signal complex (DISC) [17]. Pro-caspase-8 is then cleaved and caspase-8 is released, which in turn activates other caspases and induces an apoptotic cascade. Other proteins can bind to FADD leading to caspase-independent cell death.

Fas and FasL, along with other death receptors and their ligands, are highly expressed in the liver [18, 19]. Human hepatocytes exposed to free fatty acids increase their expression of Fas and more readily undergo Fas-mediated apoptosis *via* a mechanism that is still unknown [20]. Expression of Fas and FasL on liver cells correlates with disease severity as determined by immunohistochemistry of the normal liver, steatotic liver and NASH [21].

In adipose tissue, the roles of Fas and FasL are still being elucidated. It has been shown that human preadipocytes undergo apoptosis upon exposure to FasL and that mature murine adipocytes express Fas [22, 23]. Moreover, Fas expression is increased in the adipose tissue of mice with genetic- and diet-induced obesity and in the adipose tissue of obese humans and obese humans with T2D relative to lean, non-diabetic controls [24]. Studies of mice that are knock-out for Fas (both systemically and in adipose tissue alone) suggest that Fas may contribute to insulin resistance and steatosis by promoting the production and secretion of pro-inflammatory cytokines (*e.g.* IL-6, Cd11b, MCP-1/CCL-2) from adipose tissue [24].

Importantly, FasL can be proteolytically cleaved to produce a shorter, soluble protein (sFasL) that then circulates as a trimer [25, 26]. sFasL generally appears to promote apoptosis [27]. Its circulating levels reflect degrees of apoptosis associated with cancer and aging [28] and liver failure [29].

Ariadne-guided analysis indicated that the production of sFasL is boosted in adipose tissue of obese individuals with NAFLD. Taken together, these data prompted us to hypothesize that increased production of circulating sFasL may increase apoptosis in hepatocytes by binding to the membrane-bound Fas. Chronic exposure of the liver to signals triggering inflammation and apoptosis may then promote fibrogenesis as part of the liver’s healing response.

REFERENCES

[1] Carr MW, Roth SJ, Luther E, Rose SS, Springer TA. Monocyte chemoattractant protein 1 acts as a T-lymphocyte chemoattractant. *Proc Natl Acad Sci U S A* **1994;** *91*:3652-6.

[2] Ashida N, Arai H, Yamasaki M, Kita T. Distinct signaling pathways for MCP-1-dependent integrin activation and chemotaxis. *J Biol Chem* **2001;** *276*: 16555-60.

[3] Sozzani S, Luini W, Molino M, Jilek P, Bottazzi B, Cerletti C., et al. The signal transduction pathway involved in the migration induced by a monocyte chemotactic cytokine. *J Immunol* **1991;** *147*: 2215-21.

[4] Yen H, Zhang Y, Penfold S, Rollins BJ. MCP-1-mediated chemotaxis requires activation of non-overlapping signal transduction pathways. *J Leukoc Biol* **1997;** *61*: 529-32.

[5] Yadav A, Saini V, Arora S. MCP-1: chemoattractant with a role beyond immunity: a review. *Clin Chim Acta* **2010;** *411*: 1570-9.

[6] Gerard C, Rollins BJ. Chemokines and disease. *Nat Immunol* **2001;** *2*: 108-15.

[7] Seitz M, Loetscher P, Dewald B, Towbin H, Gallati H, Baggiolini M. Interleukin-10 differentially regulates cytokine inhibitor and chemokine release from blood mononuclear cells and fibroblasts. *Eur J Immunol* **1995;** *25:* 1129-32.

[8] Katsanos GS, Anogeianaki A, Orso C, Tete S, Salini V, Antinolfi PL, et al. Mast cells and chemokines. *J Biol Regul Homeost Agents* **2008;** *22*: 145-51.

[9] Tsuboi N, Yoshikai Y, Matsuo S, Kikuchi T, Iwami K, Nagai Y et al. Roles of toll-like receptors in C-C chemokine production by renal tubular epithelial cells. *J Immunol* **2002;** *169:* 2026-33.

[10] Pype JL, Dupont LJ, Menten P, Van Coillie E, Opdenakker G, Van Damme J et al. Expression of monocyte chemotactic protein [MCP]-1, MCP-2, and MCP-3 by human airway smooth-muscle cells. Modulation by corticosteroids and T-helper 2 cytokines. *Am J Respir Cell Mol Biol* **1999;** *21:* 528-36.

[11] Gautam SC, Noth CJ, Janakiraman N, Pindolia KR, Chapman RA. Induction of chemokine mRNA in bone marrow stromal cells: modulation by TGF-beta 1 and IL-4. *Exp Hematol* **1995;** *23:* 482-91.

[12] Vernon G, Baranova A, Younossi ZM. Systematic review: the epidemiology and natural history of non-alcoholic fatty liver disease and non-alcoholic steatohepatitis in adults. *Aliment Pharmacol Ther* **2011;** *34:* 274-85.

[13] Browning LM, Krebs JD, Magee EC, Fruhbeck G, Jebb SA. Circulating markers of inflammation and their link to indices of adiposity. *Obes Facts* **2008;** *1:* 259-65.

[14] Bruun JM, Lihn AS, Pedersen SB, Richelsen B. Monocyte chemoattractant protein-1 release is higher in visceral than subcutaneous human adipose tissue [AT]: implication of macrophages resident in the AT. *J Clin Endocrinol Metab* **2005;** *90:* 2282-9.

[15] Kanda H Tateya S, Tamori Y, Kotan, K, Hiasa K, Kitazawa R et al. MCP-1 contributes to macrophage infiltration into adipose tissue, insulin resistance, and hepatic steatosis in obesity. *J Clin Invest* **2006;** *116:* 1494-505.

[16] Tateya S, Tamori Y, Kawaguchi T, Kanda H, Kasuga M. An increase in the circulating concentration of monocyte chemoattractant protein-1 elicits systemic insulin resistance irrespective of adipose tissue inflammation in mice. *Endocrinology* **2010;** *151:* 971-9.

[17] Green DR, Ferguson TA. The role of Fas ligand in immune privilege. *Nat Rev Mol Cell Biol* **2001:** *2:* 917-24.

[18] Faubion WA, Gores GJ. Death receptors in liver biology and pathobiology. *Hepatology* **1999,** *29*: 1-4.

[19] Guicciardi ME, Gores GJ. Apoptosis as a mechanism for liver disease progression. *Semin Liver Dis* **2010;** *30*: 402-10.

[20] Feldstein AE, Canbay A, Guicciardi ME, Higuchi H, Bronk SF, Gores GJ. Diet associated hepatic steatosis sensitizes to Fas mediated liver injury in mice. *J Hepatol* **2003a;** *39*: 978-83.

[21] Feldstein AE, Canbay A, Angulo P, Taniai M, Burgart LJ, Lindor KD., et al. Hepatocyte apoptosis and fas expression are prominent features of human nonalcoholic steatohepatitis. *Gastroenterology* **2003b;** *125:* 437-43.

[22] Gross K, Karagiannides I, Thomou T, Koon HW, Bowe C, Kim H et al. Substance P promotes expansion of human mesenteric preadipocytes through proliferative and antiapoptotic pathways. *Am J Physiol Gastrointest Liver Physiol* **2009;** *296:* G1012-9.

[23] Kim JY, Wu Y, Smas CM. Characterization of ScAP-23, a new cell line from murine subcutaneous adipose tissue, identifies genes for the molecular definition of preadipocytes. *Physiol Genomics* **2007b;** *31*:328-42.

[24] Wueest S, Rapold RA, Schumann DM, Rytka JM, Schildknecht A, Nov O et al. Deletion of Fas in adipocytes relieves adipose tissue inflammation and hepatic manifestations of obesity in mice. *J Clin Invest* **2010;** *120*: 191-202.

[25] Tanaka, M, Suda, T, Takahashi, T, Nagata, S. Expression of the functional soluble form of human fas ligand in activated lymphocytes. *Embo J* **1995,** *14*, 1129-35.

[26] Vargo-Gogola T, Crawford HC, Fingleton B, Matrisian LM. Identification of novel matrix metalloproteinase-7 [matrilysin] cleavage sites in murine and human Fas ligand. *Arch Biochem Biophys* **2002,** *408:* 155-61.

[27] Bajou, K, Peng, H, Laug, W. E, Maillard, C, Noel, A, Foidart, J. M., et al. Plasminogen activator inhibitor-1 protects endothelial cells from FasL-mediated apoptosis. *Cancer Cell* **2008;** *14:* 324-34.

[28] Kavathia N, Jain A, Walston J, Beamer, BA, Fedarko NS. Serum markers of apoptosis decrease with age and cancer stage. *Aging [Albany NY]* **2009;** *1:* 652-63.

[29] Singhal S, Chakravarty A, Das BC, Kar P. Tumour necrosis factor-alpha and soluble Fas ligand as biomarkers in non-acetaminophen-induced acute liver failure. *Biomarkers* **2009;** *14*: 347-53.
